# Health Tract, No. 306

**Published:** 1867-11

**Authors:** 


### HEALTH TRACT, No. 306.

### Rational Deductions.

A LITTLE Miss, just entering her teens last summer, in the western part of Pennsylvania, took twelve saucers of ice-cream and died in a few hours; —then it would be better not to take twelve saucers of ice-cream at a time.

Within a month, D. G. P., a student of Brown University, lost his life by an abscess, induced by excessive exercise in a matched game of Ball between the students of Harvard and Brown. Then let all young persons take exercise in great moderation in the heats of summer.

A young lady of Milwaukee having heard that arsenic eaten in small and increasing quantities was a great beautifier, determined to try the experiment, but not knowing what was a small dose, a fatal result was only averted by the promptitude of the family physician; —then it follows that young ladies ought not to eat rank poison for the purpose of improving their looks.

A gentleman after active exercise laid down on an ice-chest and fell asleep, waking up with a chill, ending with death by consumption after three years of great suffering;—it is very clear that he ought not to have fallen asleep on an ice-chest soon after being over-heated.

Sometime since a man went down into a well and fell dead. A brother seeing this, hastened to descend and relieve him, and he was seen to fall dead; a third brother followed, and he fell dead.— It would have been better if the brothers had stopped going into a deadly well sooner, and to have let down a hook at the end of a rope.

One of the most respected and loved of all the Princesses of the House of Hapsburgh, set her clothes on fire by treading on a match on the floor; she died in great agony, some hours afterwards; —then lucifer matches onght not to be thrown loose around.

A French general having greatly exerted himself in bringing some artillery to the top of a mountain, drank greedily of snow-water, and dropped dead; — then persons ought not to drink snow-water greedily when over-heated.

A man took refuge from a summer shower under a solitary tree in an old field; the lightning came down the tree through his body into his boot,—spoiling the boot and killing the man; — then people who don't want their boots bursted in summer showers, thus preventing them from getting home without wetting their feet—in fact, without getting home at all—ought to lie down on the ground flat on the open field if they would make it certain that the lightning should not strike them.

An editor of a magazine, after riding all day without eating, ate a hearty meal late at nigbt, retired early, went to sleep and never waked up more; then don't eat heartily late at night; and better still, don't eat at all after sun-down, if you want to be sure of rising in the morning full of health and life.

